# Audio-Visual Perception System for a Humanoid Robotic Head

**DOI:** 10.3390/s140609522

**Published:** 2014-05-28

**Authors:** Raquel Viciana-Abad, Rebeca Marfil, Jose M. Perez-Lorenzo, Juan P. Bandera, Adrian Romero-Garces, Pedro Reche-Lopez

**Affiliations:** 1 University of Jaén, Multimedia and Multimodal Processing Group, Polytechnic School of Linares, University of Jaén Alfonso X El Sabio, 28, 23700, Linares, Spain; E-Mails: jmperez@ujaen.es (J.M.P.-L.); pjreche@ujaen.es (P.R.-L.); 2 Dpto. Tecnología Electrónica, University of Málaga, Campus de Teatinos - 29071 Málaga, Spain; E-Mails: rebeca@uma.es (R.M.); jpbandera@uma.es (J.P.B.); argarces@uma.es (A.R.-G.)

**Keywords:** multimodal perception, bio-inspired attention mechanism, human-robot interaction

## Abstract

One of the main issues within the field of social robotics is to endow robots with the ability to direct attention to people with whom they are interacting. Different approaches follow bio-inspired mechanisms, merging audio and visual cues to localize a person using multiple sensors. However, most of these fusion mechanisms have been used in fixed systems, such as those used in video-conference rooms, and thus, they may incur difficulties when constrained to the sensors with which a robot can be equipped. Besides, within the scope of interactive autonomous robots, there is a lack in terms of evaluating the benefits of audio-visual attention mechanisms, compared to only audio or visual approaches, in real scenarios. Most of the tests conducted have been within controlled environments, at short distances and/or with off-line performance measurements. With the goal of demonstrating the benefit of fusing sensory information with a Bayes inference for interactive robotics, this paper presents a system for localizing a person by processing visual and audio data. Moreover, the performance of this system is evaluated and compared via considering the technical limitations of unimodal systems. The experiments show the promise of the proposed approach for the proactive detection and tracking of speakers in a human-robot interactive framework.

## 1. Introduction

One of the main applications of robotics is to serve as assistive agents in everyday human activities. Indeed, within the last two decades, there has been an intensive effort in researching and implementing what is widely known as service robots. Some examples are found in psychological therapies, rehabilitation, education or just to serve as a recreational partner. Most of the main outcomes of research carried out upon advance human computer interaction techniques have been extrapolated to human robot interaction. As pointed out in [1], the recent merger between humanistic and engineering approaches for the enrichment of human life has become a starting point for new developments in the 21st century.

Embodied cognition is built on the idea that the robot will acquire cognitive representations through physical interaction with and exploration of the outer world. To this end, cognitive robots are currently equipped with advanced and multiple sensors. Moreover, in order to allow the correct interaction between robots and people, the broad volume of data delivered by the different types of sensors must be processed and analyzed at a high enough speed [2]. Multi-modal perception and selective attention emerge as mechanisms to deal with these issues [3] and to guide and constrain social interaction [4].

Before reacting to human actions, service or social robots may focus their attention on people depending on their perception and on the task they have to perform. Following this line of research, this paper evaluates the benefits of fusing audio and visual information in an attentional framework, so as to track one speaker in a non-controlled environment and in an interactive way. The use of more than one sensory modality promotes an increase of robustness in the observation and characterization of the robot's surroundings. Different types of sensors allow for the undermining of their individual constraints, and usually, their fusion boosts the performance obtained by adding individual sensor benefits.

Tracking a speaker has been one of the main applications for incorporating audio information within a robot's attentional mechanism. Most of the sound source localization techniques of computational auditory scene analysis in voice interactive applications (video-conference, speakers discrimination, speech recognizers) have recently been used for tracking purposes within the robotic field ([5,6]). They are usually based on the time difference of arrival (TDOA) and spectral analysis.

This paper provides a proposal for implementing a perception module capable of directing the behavioral responses of a robotic head. The perception module is built on a multimodal sensor based on a novel approach for the fusion of visual and audio sensors inside the same egocentric spatial configuration. The developed multimodal sensor combines the strength of visual and audio cues by following a bio-inspired and simple approach. This bio-inspired approach is based on three aspects: audio cues are processed based on a binaural algorithm by using just two microphones; the visual perception is built upon a system based on driving the segmentation process by the knowledge of the task; and the information from these two perception systems are fused based on Bayes inference, by using previous knowledge or experiences obtained from a training phase. Indeed, the short amount of training required is an important feature of the system, as it can be run each time the robot has to interact in a different environment.

The main contributions of this work are the following:

- Improving the consistency of the visual system (Section 3) by means of a probabilistic description of the position of persons. This description is made by a histogram called the video evidence (Section 4.1).
- Comparing the performance of the visual localization system with the OpenCV face-detector for a wide variety of conditions (changes in light, clutter backgrounds and occlusions). Face-detectors, such as the one of OpenCV library, are commonly used for tracking purposes within the robotic field. For this reason, the OpenCV face-detector is chosen as a reference to compare the visual localization system.
- Evaluating the auditory perception mechanism using binaural cues with an interactive speaker and in a real environment. The auditory system designed is described in Section 4.2, and it is based on computing the probability density of the angles of arrival estimated by the generalized cross-correlation framework (GCC)-PHAT (phase transform) method with two microphones. The GCC-PHAT algorithm is briefly reviewed in Section 3.2.
- Proposing a new approach for fusing visual and auditory cues to build a multimodal sensor. The fusion procedure, based on a Bayesian inference schema, is described in Section 4.
- Assessing the benefits on the performance of a multimodal sensor compared to unimodal ones.

Our proposal can be considered as an integration and continuation of previously published papers [7–9].

Main studies related to the design of a audio-visual perception system are reviewed in Section 2. The procedure, apparatus and the results obtained from the evaluation of this perception system are described in Section 5. Finally, Section 6 draws the conclusions and presents ongoing work.

### 2. Background

Recent work in multi-modal perception research suggests that sensor information should be integrated from the ground up, thus making integration an open issue to investigate. Previous studies have provided remarkable results in this direction [5], but they also highlight the existing difficulties. In particular, multimodal integration should consider two simple facts:

- Everything is uncertain.
- At any time, only partial knowledge of the scene is perceptually available.

To tackle these facts, several researchers have recently proven that Bayesian methods can be successfully employed [10]. Within the framework of the Bayesian coding hypothesis [11], we will extend these works by applying the Bayesian model to integrate different types of data (audio and vision) and to integrate top-down and bottom-up information in the visual streams.

One of the first studies evaluating real-time auditory and visual tracking was described in Nakadai *et al.* [12]. They presented a review of similar previous attempts based on simulations and pointed out that auditory processing and integrated perception were still based on immature technology, as well as lacking in real-time processing. Their solution was based on creating auditory, visual and associated streams in order to drive the attention control. A top-down configuration based on exploiting the proposed methods of both face and speech recognition was highlighted as a possible solution to overcome changes in the environment conditions (lighting, reverberation, noise sources, *etc.*), but to the detriment of real-time processing.

Bayesian networks are considered to be a powerful tool for information fusion. Following this approach, Asano *et al.* [13] modeled the joint probability distribution of audio and video sensors with the goal of inferring their activation co-occurrence, which was associated with the detection of a speaker. Visual tracking was based on background subtractions and audio tracking on the MUSIC algorithm. They designed this system as part of a robust speech recognizer that was able to deal with the presence of environmental noise and interferences. Thus, the experiments were made in a controlled environment limited to 60° around the tracking system and with a static configuration of speakers. They also presented one off-line experiment [14], updating their approach to a dynamic changing situation, by using a model-based approach. They evaluated the system mounted on humanoid HRP-2 to track one moving person in a restricted area.

As a step forward and by using only two microphones in order to lower computational demand, Tasaki *et al.* [15] integrated different selected sensory cues (touch, visual- skin or face-detector- and audio sensors) to interact with people based on the rules of proxemic behavior. This perception system was incorporated in the humanoid robot, SIG-2. They concluded that the integration of all the sensory modalities must be reliable.

Due to the limitations of previous works on dealing with dynamically changing environments, new approaches use spatial maps built upon active multisensory perception. In particular, Koene *et al.* [3] describes the general architecture of a robot perceptual system inspired by the superior colliculus of primates. As part of this architecture, Trifa *et al.* [16] evaluated different source localization algorithms with recordings made in a real environment through a biologically inspired auditory system mounted on a humanoid robot. In particular, they tested methods GCC, PHAT, MODD (information theory) and COCH (gamma tone filter bank imitating the cochlear system). The experimental set-up considered different noise conditions, but attended only to a one source position. Their results highlighted the method, PHAT, as the one with the greatest accuracy and MODD as the most reliable. The implemented system allowed the robot to orient its head and eyes so that it could focus its attention on audio and/or visual stimuli.

The system developed by Ferreira *et al.* ([5,17]) includes mechanisms for gaze/attention shifts (where saliency is a function of contrast), as well as a top-down guided search for stimuli that match certain object properties. They built a short-term memory as part of the top-down process based on the use of a Bayesian framework for multimodal active perception. They have integrated visual, audio and proprioceptive cues in order to build a Bayesian volumetric map, which is used to infer an entropy-based exploratory behavior. The auditory system is binaural and allows multi-speaker tracking by employing the MUSIC algorithm, while the visual system employs a face-detector based on the Haar properties of the OpenCV library ([17]) or disparity maps ([5]). In spite of the high computational demand required to build-up the volumetric map, they implemented a fast computational mechanism through lossless compression and GPU processing. The experimental results presented in both studies showed the feasibility of modeling multi-person interaction with this complex model in real time, in a non-controlled visual environment (whenever the illumination or background conditions changed), but constrained by the degradation of the auditory system due to local changes in conditions. Thus, their experimental set-up has only considered one type of audio-scenario and with short distance interaction, in order to overcome the limitations of the face-detector algorithm used and the possible degradation of the auditory system.

Nickel and Stiefelhagen [18] also described a similar approach to build a joint attention map of surroundings to track multi-person in real time by using a humanoid stereo head. In order to overcome the typical problems of occlusion or background clutter, whilst maintaining real-time rates, researchers have proposed the use of a particle-filter based on the democratic integration of fast visual cues for 3D tracking. In addition to the visual attention tracker, an audio tracker was implemented by applying the PHAT algorithm on an array of microphones. Reported experiments were made by reducing the number of features and over a video sequence recorded under the control of a human operator. The authors concluded that there was a need to optimize the number of parameters involved. A particle filtering model has been also employed [6] to integrate information sensed by thermal and distance cameras with audio information. The performance of this method has been evaluated by comparing separately an audio-based localization system, a visual-based localization system and an audio-visual tracking system. Their study probed the robustness of the last method for tracking the azimuth direction of one speaker.

Bottom-up attention has been widely studied in cognitive fields, and many computational models have been built according to the structure of the feature integration theory [19] and the guided search model [20]. Furthermore, during the last decade, several approaches came up to model the saliency with computational and mathematical methods that are mostly less biologically motivated [21]. Specifically, information theory has recently entered into the field of bottom-up saliency estimation ([21–23]. Low-level attention originated as a necessary mechanism to constrain the computational complexity of perception ([24]), by highlighting those image features or regions that have a higher probability of being relevant. Saliency maps are probably the most popular means of evaluating visual scenes in a way that approximates human early vision [25].

Fast, simple methods for accurate acoustic source localization are commonly used in robotics. Moreover, they are usually based on a biologically inspired configuration by employing binaural information acquired through just two microphones. Other approaches also exist based on using more than two microphones to enrich the information obtained and the estimation accuracy, but at the expense of higher complexity and computational cost. Most of the algorithms used for multi-speaker tracking have been based on methods, such as MUSIC, which requires prior knowledge of the signal environment in order to work properly. For example, if the number of sources is incorrectly estimated, the performance of these algorithms will deteriorate significantly.

In the study presented here, audio cues have been treated as a source of fast perceptual information that helps to overcome limitations imposed by the visual system and that allows fast attention drifts to unexpected events. On the other hand, fine location estimation is left to the visual system. That is to say, the multimodal sensor implemented uses the audio inputs combined with visual ones as a first gross filter, and its outputs are later refined with the higher resolution of visual segmentation. In particular, the method used belongs to the generalized cross-correlation framework (GCC), which consists of summing the signals for every possible delay between two microphones and then selecting the delay for which the sum is maximal as an estimate of the TDOA. Moreover, the probabilistic approach followed may be further exploited for multi-speaker tracking purposes.

### 3. Visual and Audio Tracking Systems

This section describes how the visual and audio cues are processed in order to be included in the attention model.

#### 3.1. Visual Saliency Computation

The visual scene is encoded by using the bounded irregular pyramid (BIP) [7], which is built by means of a perceptual segmentation approach. Perceptual segmentation can be defined as the process that allows the organizing of low-level image features into higher level relational structures. Handling such high-level features instead of image pixels offers several advantages, such as the reduction of the computational complexity of further processes. In addition, this approach provides an intermediate level of description (shape, spatial relationships) for data, which is more suitable in object recognition tasks.

Perceptual segmentation approaches typically integrate a pre-segmentation stage with a subsequent perceptual grouping stage. Basically, the first stage conducts the low-level definition of segmentation as a process of grouping pixels into homogeneous clusters; meanwhile, the second stage performs a domain-independent grouping of the pre-segmentation regions, which is mainly based on properties, like the proximity, similarity, closure or continuity. A detailed description of the employed perceptual segmentation approach can be found in [26]. In the proposed pre-segmentation stage, nodes are grouped using a color similarity criteria. In the proposed perceptual grouping stage, more complex similarity criteria, based on color, edge and disparity, have been used.

Once the visual scene has been encoded via using the BIP and the set of proto-objects has been extracted by means of the indicated perceptual segmentation approach, the saliency of each proto-object must be computed. In the proposed system, this visual saliency is the linear combination of multiple basic features. Specifically, four low-level features have been selected: color and intensity contrast, disparity and skin color.

The color contrast of a proto-object, *i*, is computed as the mean color gradient, *MCG_i_*, along its boundary to the neighbor regions:

(1) $$MCG_{i}=\frac{S_{i}}{b_{i}}\sum_{j\in N_{i}}b_{ij}\cdot d\left(<C_{i}>,<C_{j}>\right)$$

being *b_i_* the perimeter of *i*, *N_i_* the number of regions that are neighbors of *i*, *b_ij_* the length of the perimeter of region *i* in contact with region *j*, *d*(< *C_i_* >,< *C_j_* >) the color distance between the color mean values, < *C* >, of the regions, *i* and *j*, and *S_i_* the mean saturation value of region *i*. The use of *S_i_* in the *MCG* avoids that the color proto-objects with low saturation (gray regions) obtain a higher value of color contrast than pure color proto-objects. The problem is that white, black and pure gray regions are totally suppressed. To take into account these regions, the luminosity contrast is computed. The luminosity contrast of a proto-object, *i*, is the mean luminosity gradient, *MLG_i_*, along its boundary to the neighbor regions:

(2) $${\text{MLG}}_{i}=\frac{1}{b_{i}}\sum_{j\in N_{i}}b_{ij}\cdot d\left(<I_{i}>,<I_{j}>\right)$$

being < *I_i_* > the mean luminosity value of *i*.

In order to obtain an accurate disparity map, *D_i_*, the small vision system (SVS) provided by Videre Design (www.videredesign.com) has been used in this work. SVS is a set of library functions that implement the stereo algorithms and compute the disparity using a correlation-based scheme. The disparity obtained by SVS is averaged inside each proto-object, *i*, in order to obtain a unique value of disparity, *D_i_*, for them.

Skin color (*SK_i_*) has been integrated as an independent feature in order to distinguish locations where a human can be placed. In order to determine if a proto-object is skin colored, the skin chrominance model proposed by Terrillon and Akamatsu [27] has been used. If, following this model, a proto-object results in being skin-colored, then it is marked with a value of 255 in the skin color map, *SK*. In any other case, it is set to zero.

Finally, the saliency, *Sal_i_*, of each proto-object is computed by combining the previous features into a single value using a weighted normalized summation:

(3) $${\text{Sal}}_{i}=\lambda_{1}\cdot {\text{MCG}}_{i}+\lambda_{2}\cdot {\text{MLG}}_{i}+\lambda_{3}\cdot D_{i}+\lambda_{4}\cdot SK_{i},\sum_{i=1}^{4}\lambda_{i}=1$$

being {λ}*_i_*_=1…4_ the weights associated to each low-level feature, the values of which are set depending on the current task to execute in the attentive stage. Using these weights, top-down information is included in the attention system. In this study, these values have been experimentally selected for the proper tracking of a person.

#### 3.2. Acoustic DoA Estimation

The audio tracking system implementation requires an algorithm that allows one to estimate the direction of arrival (DoA) of the signal from the speaker. In our proposal, the acoustic DoA estimation is implemented by means of the PHAT (phase transform) algorithm, which belongs to the generalized cross-correlation (GCC) framework. Among the GCC methods, the PHAT algorithm has been recently highlighted [8] as a very robust method for uncorrelated noise and reverberation through experiments in real environments. Besides, it has been pointed out as a reliable algorithm for multi-source detection by adding clustering methods ([28,29]).

**Figure 1.**
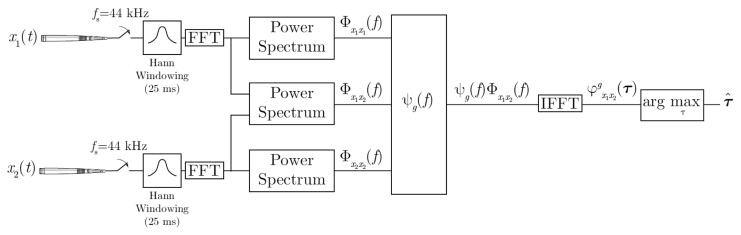
Block diagram for PHAT (phase transform).

As shown in Figure 1, the generalized cross-correlation, $\varphi_{x_{1}x_{2}}^{g}(\tau )$, is expressed in terms of the cross power spectral density function, Φ*_x_*__1__*_x_*__2__ (*f*), as described in Equation (4):

(4) $$\varphi_{x_{1}x_{2}}^{g}(\tau )=\int_{-\infty }^{\infty }\psi_{g}\left(f\right)\Phi_{x_{1}x_{2}}(f)e^{j2\pi f\tau }df$$

being *x*_1_ and *x*_2_ the signals captured at both microphones. *ψ_g_*(*f*) is a weighting function, and in particular, the PHAT weighting is computed as:

(5) $$\psi_{g}(f)=\frac{1}{\left|\Phi_{x_{1}x_{2}}(f)\right|}$$

**Figure 2.**
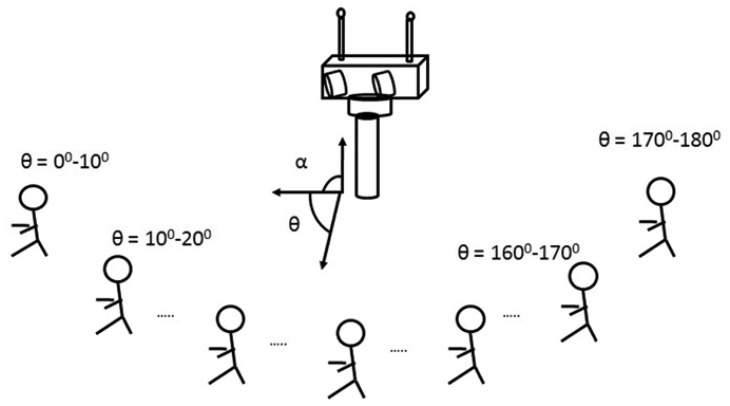
Reference system for egocentric azimuth and elevation angles computed through visual and audio systems.

The Audio-Process service implements PHAT with two omnidirectional microphones and with a sampling frequency of 44.1 KHz. Every 12.5 ms TDOA between the two microphones is computed, and therefore, the azimuth angle (*θ*) refers to the initial position of the robot's head, as can be seen in Figure 2. The direction of arrival is estimated with analysis frames of 25 ms, windowed with a Hann window with a 50% overlap. As described in Figure 1, the cross-power spectral-density function is obtained in each window using 1,024 sample Fourier transforms via FFT, and the cross-correlation is computed with its corresponding inverse Fourier transform via IFFT. A parabolic interpolation stage is also done to search the maximum peak in the correlation function with a higher resolution. For each analysis window, the TDOA (*τ̂*) is estimated and the corresponding angle computed as:

(6) $$\hat{\theta }=\arccos\left(\frac{c\cdot \hat{\tau }}{d}\right);\text{being}\hat{\tau }=\arg\underset{\tau }{\max}\varphi_{x_{1}x_{2}}^{g}(\tau )$$

being *c* = 343 m/s (for a lab temperature of approximately 20 °C) the speed of sound and *d* the separation between microphones (13.5 cm).

### 4. Bio-Inspired Multimodal Sensor Implementation

As has been stated, the main goal of this work is to implement a robust sensor by integrating visual and audio cues in an attentional mechanism. Audio detection may act to outperform the detection range of speakers or noisy people, as well as to improve the performance of a bio-inspired visual tracking system under changes of illumination conditions and backgrounds. Moreover, visual information adds to the capacity of being able to obtain the elevation angles from the robotic head and a higher tracking resolution. The integration developed with the information obtained from the segmentation process (proto-objects) and the DoA estimation of sound (azimuth angles of the speaker or a noisy person) are described in the following subsections.

**Figure 3.**
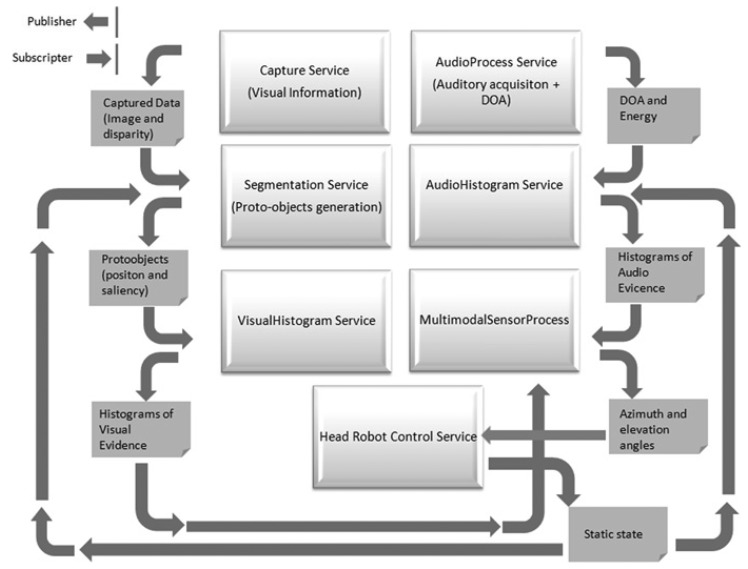
Services structure of the implementation of the multimodal sensor.

The complete system consists of several services that exchange information using Nerve, a lightweight middleware for robotics built upon the DDS (Data Distribution Service) specification. As described in [9], this middleware makes use of several design patterns available in the ACE toolkit and guarantees both a high-performance and the requirement of quality of service for real-time scenarios. Figure 3 shows the different services involved in the robot's head movement and the multimodal sensor implementation. The main features of the core modules are detailed in the following sections.

#### 4.1. Proto-Objects Localization Histograms

As explained in Section 3.1, the segmentation module provides a set of proto-objects featured with their respective positions in the image and their saliency. Considering the camera model, these proto-objects are reorganized depending on their position (center transformation) expressed in azimuth and elevation angles referring to the initial robotic head position. For every 10 segmented images (around 1 s), a histogram was built based on the azimuth angle of the proto-objects and their saliency value. To obtain a more accurate and stable estimation of the person's position, proto-objects placed in angles with high saliency are given a greater weight in the accumulating process. In addition, during the first second of recording, the average saliency value (ranging from zero to 255) of the surrounding environment is computed and established as a threshold for the saliency. Thus, the proto-objects with a saliency below this threshold are not considered and, therefore, not computed within the histogram accumulation.

**Figure 4.**
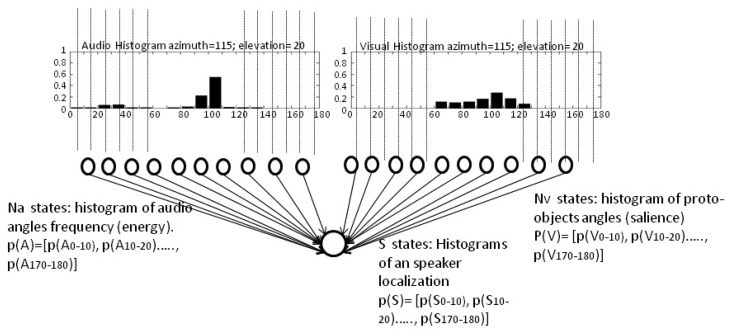
The Bayesian network used to infer the speaker localization based on the histograms of the audio and visual evidences.

The histogram built is called the video evidence, and it defines the probability of having a human in a specific region. An example of this histogram is shown in Figure 4. It shows the probability function estimation of the visual evidence of having a person placed between 0° and 180° around a robotic head, with an equi-width histogram of 10°, that is to say, with 18 bins. The histogram values are also treated as a vector **p**(*V*) = [*p*(*V*_0−10_),*p*(*V*_10−20_), …,*p*(*V*_170−180_)]*^t^* that represents the probability of having a person between those ranges. To simplify, these ranges are referred to as Θ*_i_* with *i* = 1..18, where the *i*-*th* range represents a localization between angles (*i* – 1)·10° and (*i*)·10°. Thus, from now on, the visual histogram is represented as: **p**(*V*) = [*p*(*V*_Θ_1__),*p*(*V*_Θ_2__), …,*p*(*V*_Θ_18__)]*^t^*

For example, the data shown in Figure 4 represents a higher probability of having a person in the bin, Θ_10_, that is to say, within the range 100°–110°.

#### 4.2. Acoustic DoA Histograms

In order to make a robust estimation of the DOA angles audio sources, a probabilistic treatment is made by means of a histogram construction. This histogram was computed via accumulating the DoA angles (see Section 3.2) estimated in 80 windows of 25 ms. At the beginning of the experiments, the energy level of ambient noise is computed for 1 s, with the purpose of selecting only the frames with a certain level of voice energy. Thus, the estimations obtained in windows with an energy level under the previously established threshold based on that initial energy level were discarded. The threshold can be configured depending on the environment conditions, but in these experiments, it was established as the level of energy that allows detecting a speaker talking low, at 4 m.

The histogram computed can be seen in Figure 4. It is represented as the vector: **p**(*A*) = [*p*(*A*_0−10_),*p*(*A*_10−20_), …,*p*(*A*_170−180_)]^*t*^. Following the same nomenclature described in the previous section, it is represented as: **p**(*A*) = [*p*(*A*_Θ_1__),*p*(*A*_Θ_2__), …,*p*(*A*_Θ18_)]^*t*^.

#### 4.3. Fusion of Audio and Video Evidences

##### 4.3.1. Fusion

The integration of audio and video information is based on the inference developed over a Bayesian network. This network is built to model the joint probability distribution of two variables representing the audio (estimated DoA angles of possible sound sources) and visual events (locations of possible proto-objects representing a speaker or a noisy person). Based on this joint probability, the variable to evaluate corresponds with the probability of having a person who speaks or makes noise on a specific range of angles.

Figure 4 shows the network used and the possible states of the three variables considered. As can be seen, the audio information is represented by *Na* audio nodes, corresponding with 18 possible angle ranges or bins of auditory source detections. In the same way, vision information is represented by *Nv* nodes that are related with 18 angles ranges of observations where the segmented proto-objects associated with a person could be placed. Therefore, *Ns* corresponds with the 18 possible regions where auditory and visual sensors can be activated. The inference on this network is only applied when sound is detected. In the case that the audio energy level does not indicate the presence of a person, just the visual information is used to track them.

Each of the vectors in Figure 4 represents the histograms of the probability functions of variables considered. Although it is not indicated in the figure, these histograms change as a function of time when the sensor updates the auditory and visual evidence, via creating the sensor evidence, **p**(X), as:

(7) $$p(X)=\left(\begin{array}{c} p(A)\\ -\\ p(V) \end{array}\right)$$

The inference over the Bayes network allows computing the histogram of the speaker's position **p**(*S*) = *[p*(*S*_Θ_1__),*p*(*S*_Θ_2__), …,*p*(*S*_Θ_18__)]*^t^*, and the estimation of the azimuth angle is then obtained as:

(8) $$\hat{\Theta }=\arg\underset{\Theta_{i}}{\max}P\left(S/X\right)\cdot p(X)$$

##### 4.3.2. Inference Process

As explained in the previous section, the state of the output node or the speaker is estimated through an inference process carried out on a Bayesian network. Thus, the state of *S* can be determined by estimating its conditional probability (the “a posteriori” probability is the conditional probability assigned after the relevant evidence is considered), which is referred through the matrix **P**(*S*/*X*) = (**P**(*S*/*A*)|**P**(*S*/*V*)).

It is assumed that once *S* is fixed to a certain state, the distributions of audio and visual observations become nearly independent, because once the effect of the common cause is removed (a speaker placed on a specific position detected through the cameras and the microphones) from the distribution, only the independent noisy fluctuation of observations remains. That is to say, ideal sensors would show in the histograms of Figure 4 just one peak around 100°. However, the auditory sensor is also affected by inherent noise (coherent or incoherent noise) and the acoustic properties of the room (reverberation, scattering, *etc.*), which are independent of the inherent noise of the vision sensor, such as objects with high saliency, room background, luminosity changes, *etc*.

Based on this assumption, the conditional probability or *a posteriori* probability can be obtained from this expression:

(9) P(S/X)⋅p(X)=P(S/X)⋅p(S)

The prior distribution of *S* (it describes the *a priori* beliefs about the speaker position) is assumed to be uniform. Therefore, P(*S_i_*) = 1/Ns.

Once this conditional probability is calculated, the histogram of the **p**(S) distribution function can be computed by applying the Bayes theorem to the probabilities of the evidence (**p**(V) and **p**(A)), which have been obtained from the audio and visual sensors every 1 s. Then, the region where the speaker or a noisy person is placed can be estimated through Equation (8).

##### 4.3.3. Training Phase

The conditional probability table or likelihood table (which describes how the sensor measurement depends on the true value, the background information and the sensor status) is estimated from training samples. Thus, the performance of the multimodal sensor is based on a training phase, in which a person speaks for a few seconds while being placed in 18 different positions corresponding with angles ranging from 0° to 180° (see Figure 2). The robot's head was kept in the initial position during all of this training phase. From each step, auditory and visual modules reported histograms that were used to build each row of the following conditional probability matrix **P**(*X*/*S*) = (**P**(*A*/*S*) |**P**(*V*/*S*)), where the conditional probability matrix corresponding to the audio histograms measured at each speaker location is:

(10) $$P\left(A/S\right)=\left(\begin{array}{ccccc} p\left(A_{\Theta_{1}}/S_{\Theta_{1}}\right) & p\left(A_{\Theta_{2}}/S_{\Theta_{1}}\right) & ‥ & ‥ & p\left(A_{\Theta_{18}}/S_{\Theta_{1}}\right)\\ p\left(A_{\Theta_{1}}/S_{\Theta_{2}}\right) & p\left(A_{\Theta_{2}}/S_{\Theta_{2}}\right) & ‥ & ‥ & p\left(A_{\Theta_{18}}/S_{\Theta_{2}}\right)\\ ‥ & ‥ & ‥ & ‥ & ‥\\ ‥ & ‥ & ‥ & ‥ & ‥\\ p\left(A_{\Theta_{1}}/S_{\Theta_{18}}\right) & p\left(A_{\Theta_{2}}/S_{\Theta_{18}}\right) & ‥ & ‥ & p\left(A_{\Theta_{18}}/S_{\Theta_{18}}\right) \end{array}\right)$$

and the conditional probability matrix corresponding to the visual histograms measured at each speaker location is:

(11) $$P\left(V/S\right)=\left(\begin{array}{ccccc} p\left(V_{\Theta_{1}}/S_{\Theta_{1}}\right) & p\left(V_{\Theta_{2}}/S_{\Theta_{1}}\right) & ‥ & ‥ & p\left(V_{\Theta_{18}}/S_{\Theta_{1}}\right)\\ p\left(V_{\Theta_{1}}/S_{\Theta_{2}}\right) & p\left(V_{\Theta_{2}}/S_{\Theta_{2}}\right) & ‥ & ‥ & p\left(V_{\Theta_{18}}/S_{\Theta_{2}}\right)\\ ‥ & ‥ & ‥ & ‥ & ‥\\ ‥ & ‥ & ‥ & ‥ & ‥\\ p\left(V_{\Theta_{1}}/S_{\Theta_{18}}\right) & p\left(V_{\Theta_{2}}/S_{\Theta_{18}}\right) & ‥ & ‥ & p\left(V_{\Theta_{18}}/S_{\Theta_{18}}\right) \end{array}\right)$$

From matrix **P**(X/S), the *a posteriori* matrix **P**(*S*/*X*) = (**P**(*S*/*A*)\**P**(*S*/*V*)) is computed as:

(12) $$P\left(S/A\right)=\left(\begin{array}{ccccc} \frac{p\left(A_{\Theta_{1}}/S_{\Theta_{1}}\right)}{Z_{1}} & \frac{p\left(A_{\Theta_{2}}/S_{\Theta_{1}}\right)}{Z_{2}} & ‥ & ‥ & \frac{p\left(A_{\Theta_{18}}/S_{\Theta_{1}}\right)}{Z_{18}}\\ \frac{p\left(A_{\Theta_{1}}/S_{\Theta_{2}}\right)}{Z_{1}} & \frac{p\left(A_{\Theta_{2}}/S_{\Theta_{2}}\right)}{Z_{2}} & ‥ & ‥ & \frac{p\left(A_{\Theta_{18}}/S_{\Theta_{2}}\right)}{Z_{18}}\\ ‥ & ‥ & ‥ & ‥ & ‥\\ ‥ & ‥ & ‥ & ‥ & ‥\\ \frac{p\left(A_{\Theta_{1}}/S_{\Theta_{18}}\right)}{Z_{1}} & \frac{p\left(A_{\Theta_{2}}/S_{\Theta_{18}}\right)}{Z_{2}} & ‥ & ‥ & \frac{p\left(A_{\Theta_{18}}/S_{\Theta_{18}}\right)}{Z_{18}} \end{array}\right)*1/N$$

where $\text{N}=\text{Na}+\text{Nv};Z_{i}=p\left(A_{\Theta_{i}}\right)=1/Ns*\sum_{k=1}^{Ns}p\left(A_{\Theta_{i}}/S_{\Theta k}\right)\;\text{for}\;i=1‥\text{Na}.$

(13) $$P\left(S/V\right)=\left(\begin{array}{ccccc} \frac{p\left(V_{\Theta_{1}}/S_{\Theta_{1}}\right)}{Y_{1}} & \frac{p\left(V_{\Theta_{2}}/S_{\Theta_{1}}\right)}{Y_{2}} & ‥ & ‥ & \frac{p\left(V_{\Theta_{18}}/S_{\Theta_{1}}\right)}{Y_{18}}\\ \frac{p\left(V_{\Theta_{1}}/S_{\Theta_{2}}\right)}{Y_{1}} & \frac{p\left(V_{\Theta_{2}}/S_{\Theta_{2}}\right)}{Y_{2}} & ‥ & ‥ & \frac{p\left(V_{\Theta_{18}}/S_{\Theta_{2}}\right)}{Y_{18}}\\ ‥ & ‥ & ‥ & ‥ & ‥\\ ‥ & ‥ & ‥ & ‥ & ‥\\ \frac{p\left(V_{\Theta_{1}}/S_{\Theta_{18}}\right)}{Y_{1}} & \frac{p\left(V_{\Theta_{2}}/S_{\Theta_{18}}\right)}{Y_{2}} & ‥ & ‥ & \frac{p\left(V_{\Theta_{18}}/S_{\Theta_{18}}\right)}{Y_{18}} \end{array}\right)*1/N$$

where $\text{N}=\text{Na}+\text{Nv};Y_{i}=p\left(V_{\Theta_{i}}\right)=1/Ns*\sum_{k=1}^{Ns}p\left(V_{\Theta_{i}}/S_{\Theta k}\right)\;\text{for}\;i=1‥\text{Nv}.$

##### 4.3.4. Angle Determination of Speakers

As previously described, if the audio evidence indicates that there is a speaker or a noisy person, the fusion of the evidence is made with the Bayesian network described in Section 4.3.3. in order to obtain the region with the highest probability of having a speaker. In the other case, only the vision evidence is used to detect a possible quiet person. This process of azimuth angle determination is made every 1 s by computing the speaker histogram (**p**(S)) and by extracting the estimated azimuth angle (Θ̂, see Equation (8)).

Once the azimuth region with the highest probability of having a person has been determined, if the region belongs to the actual field of view of the cameras, the structure built up by the visual localization module is explored to detect the proto-object with the highest saliency in the region. The azimuth and elevation angles of the center of this proto-object are sent to the robot's head module control. Beside updating pan and tilt values, the service that implements the head module control is also in charge of inhibiting auditory and visual sensors, while the head is moving towards the new position. This is mainly done to avoid the side effects of auditory sensitivity to motor noise and the possible incoherence of visual evidence, due to the head movement.

### 5. Experiment and Results

The benefits of the multimodal sensor are evaluated by analyzing first the output of the AudioHistogram and VisualHistogram services (see Figure 3). In the following sections, the main aspects of the experiments and results obtained are described.

#### 5.1. Apparatus

The testbed used in the experiments is based on a stereo vision and binaural set-up mounted on a motorized head. In particular, it is a Zebra (SMC-629) pan/tilt/vergence unit where a stereo-vision system is mounted using STH-MDCS from Videre Design. This system consists of two 1.3 mega-pixel, progressive scan CMOS imagers mounted in a rigid body and a 1,394 peripheral interface module, joined in an integral unit. Images are restricted to 320 × 240 pixels. The field of view of the cameras is around 65.2° for the azimuth and 51.3° for the elevation.

**Figure 5.**
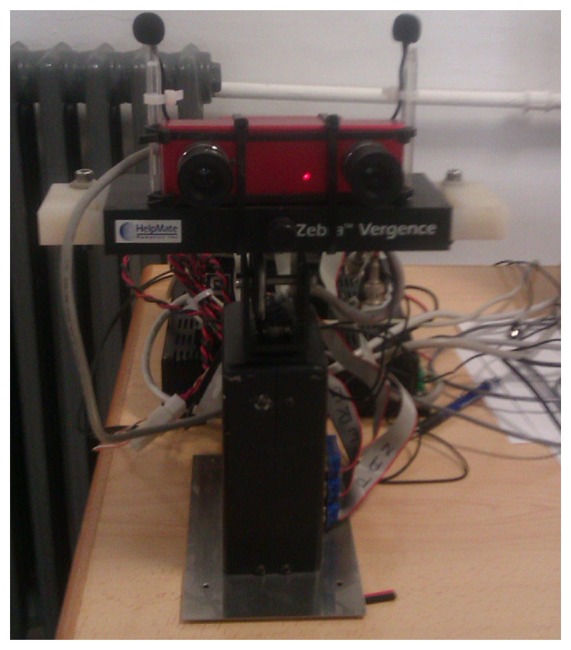
Stereo vision and binaural set-up mounted on the motorized head used for the experiments.

The binaural set-up consists of two omni-directional AKG-C417PP microphones and a M-audio USB sound-card. As shown in Figure 5, the two microphones are placed on the motorized robot with a distance between them of 13.5 cm. The multimodal sensor run on a PC (HPZ400-Intel Xeon W3565 a 2.67 GHz) using the Linux operating system. The segmentation system is able to process 320 × 240 images at 10 frames per second.

The room where the measurements were made has a volume of 248.64 m^3^. The average reverberation time is approximately 1 s, measured according to ISO 3382:1997. It is a research lab with the ambient noise of an office and typical sound sources related to computers, ceiling fan/heating systems, keyboard typing and low-level background conversations.

#### 5.2. Procedure and Metrics

The visual and auditory systems were tested attending to different conditions of light, background and ambient noise. The evaluations were made with the robotic head in a static configuration and with a person moving around it, standing at different positions and with different backgrounds (the robot's head was manually moved). As a measurement, the visual and audio histograms were recorded. In addition, snapshots taken with the left camera of the stereo-vision setup were saved in a single-channel image. In these images, the detection performed by the tested system is drawn as a red circle. Furthermore, the faces estimated by a face-detector (Haar-like features implementation of the OpenCV library) are drawn with blue circumferences. This information was used in order to highlight the possible strengths and limitations of these methods compared with using a common tool widely used for tracking people.

The multimodal sensor, that is to say, the fusion of the visual and auditory system outcomes, was tested with two different experiments. The first experiment was made in a static configuration with a speaker talking around the robot's head and the second experiment by updating the robot's head position with the inferred angles (highest probability of having a speaker), through the Head Robot Control Service (see Figure 3).

### 5.3. Results and Discussion

#### 5.3.1. Visual Localization System

As described in Section 3, the visual system was designed for a general attentional system focused as a main task on tracking speakers. Thus, the system computes the saliency of the proto-objects giving the highest value to the weight of the skin-color feature, but also considering other features useful for possible tasks where a person is not necessarily involved. The saliency weights have been configured for these tracking purposes to the following values: 0.15 for the color contrast, 0.15 for the intensity contrast, 0.3 for disparity and 0.4 for the skin color, based on experimental results reported in [25].

**Figure 6.**
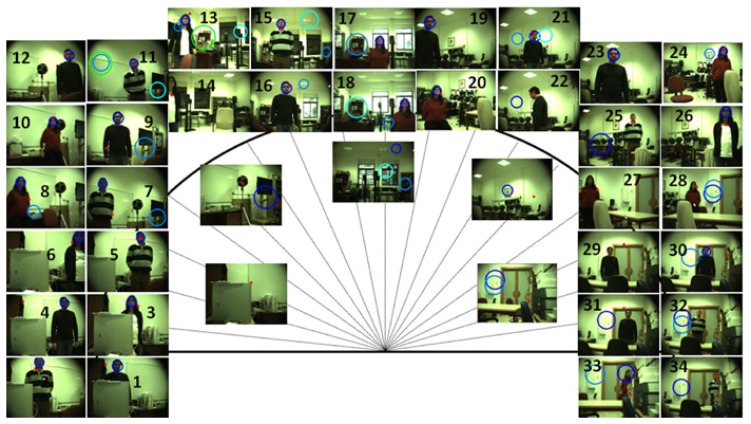
Visual localization system performance compared to a face-detector algorithm in five different backgrounds displayed in the images placed inside the circumference. The red circle on the image represents the visual system estimation. Blue circumferences show OpenCV face-detector estimations.

In order to test the histograms robustness built upon the proto-objects in a real scene with different cluttered backgrounds, the system has been tested by placing the robot's head in front of five different backgrounds and with different lighting conditions (day and night time). Figure 6 shows a red mark placed at the average position (azimuth and elevation angles) of the proto-object with the highest saliency for the images computed. The OpenCV face-detector outcomes are also shown in this figure with blue spheres for comparison purposes. The experiment consisted of tracking one person when he/she was standing at different azimuth angles and at distances ranging from 1 to 4 m from the robot's head.

One of the conclusions obtained from the different tests performed was the degradation of the performance of both systems during daytime, when the objects brightness and shadows were affected by the light coming into the room through the windows.

For the first six frames (Background 1), the person was placed close to the robotic head, at a distance of approximately 1 m. As can be seen in Figure 6, the face-detector performed more accurately in all the lighting conditions and localizations. Indeed, the visual system failed its detection in Frames 1 and 4, where another object was considered as a proto-object associated with a person.

The following background (Frames 7 to 12) consisted of a white blackboard and a disrupting object (omnidirectional speaker), which was marked as a person for both systems when she/he was not visible. As can be seen in these images, the OpenCV face-detector also worked with higher accuracy, and the main difference can be seen on the elevation angle estimation.

The third background is featured by a higher luminosity for the tests made during the day (Frames 16–18). Here, the performances of the two visual systems were similar. Frame 14 shows how the face-detector was more affected by partial occlusions of the face than our visual system.

In Background 4, the person detection was more accurate for the visual localization system than for the face-detector when the person was placed at a distance further away from the robot;s head and with a clustered background or possible rotations of the face (Frames 19–26). Thus, as can be seen in Frames 22 and 25, rotations and distance seem to be a limitations for a standard face-detector, while the detection was suitable for the implemented visual system.

In Background 5 (Frames 27–34), the performance was affected negatively by the distance in the face-detector and the existence of other proto-objects marked as a possible person in the proposed system. The implemented visual localization system performed with certain accuracy for Frames 27–28, but once the lateral cartons were present on the scene (Frames 29–34), the performance degradation was evident. Furthermore, the face-detector failed in nearly all the frames with distances between 2–4 m.

Finally, as can be seen in the frames taken without a person (frames non-numbered), one of the main problems of the visual systems was the detection of false positives. Both systems detected faces or proto-objects related with a person in the five backgrounds considered; although this behavior was expected for the proposed system, as more features than just the skin color were considered for tasks without the need of having a person involved.

#### 5.3.2. Audio Localization System

**Figure 7.**
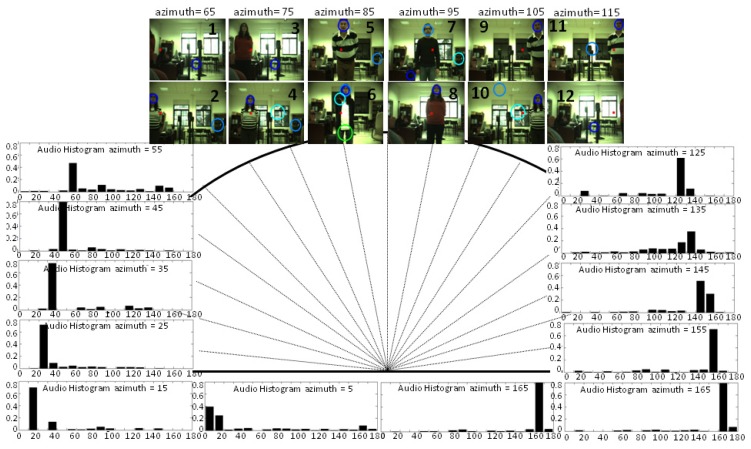
The results obtained with different speakers and noise source conditions while tracking a speaker with the audio localization system. The histograms show the evidence encountered for each detection of 1 s in the 12 angles out of the camera field of view. The images show with a red circle the audio system estimation made within the field of view area. Blue circumferences show OpenCV face-detector estimations.

Although audio processing is faster than visual processing, in order to compute a significant number of samples to create the histogram (where the number of bins is the square root of the number of samples), the number of detection ranges or histogram bins is established as 18; that is to say, in 10° intervals, with the intermediate value as the representative one. This range also corresponds with the error in direction typical in humans of about 8–10°. During the first second of the session, the ambient noise energy is computed and used as explained in Section 4. After that, a person was asked to be placed in 18 positions, talking for a few seconds, and at different distances from the robot's head (between 1 and 4 m). As shown in Figure 7, the mode of the audio histograms corresponds with the angle range where the speaker is placed in nearly all the cases. Indeed, there are usually only incorrect estimations close to the lateral bins (5°, 15°, 165°, 175°), and for the case represented in this figure, the estimation was only wrong when the speaker was located around 175°, the estimation for this case being 165°.

As explained in [8], there is a performance degradation of GCC methods employed with only two microphones close to 0° and 180°, which is limited by the frequency sampling, due to the microphones' distance and the non-lineal transformation of Equation (6). Besides, these methods deliver their best performance when the sound source is placed at 90°, that is to say, when the audio signals in both microphones are nearly identical. This is one of the advantages of having a dynamic system where the robot's head is moved to point to the detected angle, improving, therefore, the estimation of surrounding bins where there is a higher probability of finding the speaker in the subsequent seconds.

In the six locations or bins that fall into the field of view, the audio system was able to localize the speaker in angles where OpenCV had difficulties. In the lateral bins corresponding to 65° and 125° (Frames 1–2, 11–12), the audio localization system was able to identify the speaker's angle with a certain error, while the speaker's face was still out of the frame or not completely visible. When dealing with just visual information, the system must also consider the brightness changes that were encountered in Frames 3,4,7 and 8, due to the light coming from the window. In these cases, the OpenCV face-detector had difficulties correctly detecting the face and/or it detected different possible face alternatives. However, in frames where the face-detector accurately estimated the speaker's position (Frames 5,6,9,10 and 11), the audio system showed its main weakness (see Frames 9 and 11): a lower resolution and accuracy (only azimuth angles) compared with the visual system, which was able to perfectly locate the speaker's face on the frames.

Summarizing, the auditory localization system is able to properly track one person in real conditions and in interactive time. The experimental results corroborated the conclusions already obtained in a previous work [8] for the PHAT algorithm, but in off-line tests with sounds recorded in different real scenarios. In particular, PHAT was found to be the most suitable approach for conditions of 20 dB of SNR with stationary noise (fan noise) and in a scenario with a medium and high value of reverberation time.

#### 5.3.3. Audio-Visual Localization System

**Figure 8.**
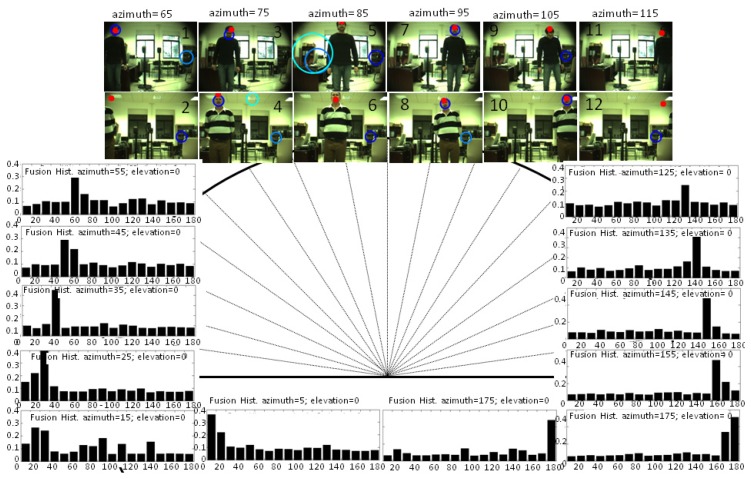
Tracking results obtained with different speakers and noise source conditions. The histograms show the evidence encountered for a detection of 1 s in different positions. The images show with a red circle the estimations of the multimodal sensor placed within the field of view. Blue circumferences show the face-detector outcomes.

The results of the multimodal sensor while tracking a speaker are shown in Figure 8 with the fixed position of the robot's head. The histograms are shown for the frames when the speaker is outside the cameras field of view. As can be seen, the mode was properly estimated in nearly all the cases. Estimations on the lateral bins or angle ranges were still not completely accurate, and for this case, the localization estimation was wrong in 165°. For the bins within the field of view, the estimation of the multimodal sensor is shown with a red circle within the corresponding snapshots, identifying the average position of the proto-object with the highest saliency. The obtained results are shown in two conditions of luminosity: day and nighttime. In all the cases, results showed a better performance of the multimodal sensor than the estimation made with OpenCV. The results were more accurate than those obtained by the visual and audio localization systems with the same backgrounds and auditory conditions. Indeed, the strength of this system is clearly evident in Frames 2, 11 and 12, where the face was not completely visible. and for Frames 5, 6 and 9, where the face-detector failed, due to uncompleted faces or rotations.

An error analysis was also made with the sensor estimations that were clearly marked as a speaker's detection. The mean square error of prediction (the difference between landmarks and angles estimated) is 0.47° with a standard deviation of 4.9°, which can be considered as a suitable value for robot-human interaction. Moreover, this error is lower for regions within the camera field of view, where the speaker is placed most of the time when the robot's head is moving.

These results were in accordance with those presented in [5] for a single speaker placed in one position. Indeed, they represented a complete reconstruction of the robot's head perception, showing the strength of fusing visual and audio cues compared to just using one of the modalities of information.

**Figure 9.**
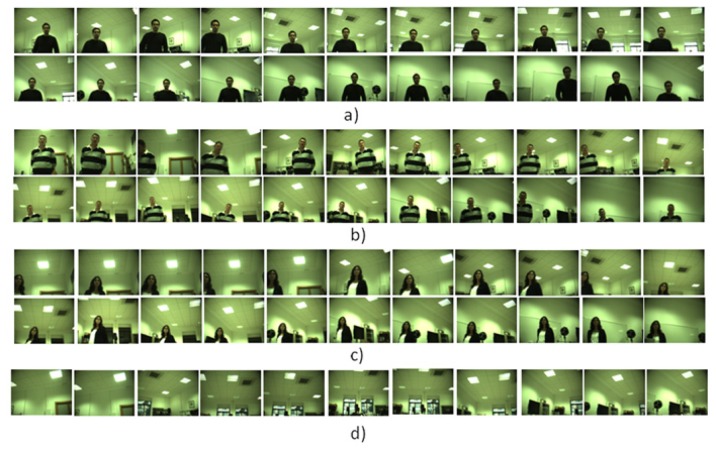
Results obtained with different speakers and noise source conditions while tracking a speaker in interactive time conditions with the multimodal sensor. The robot's head position (azimuth and elevation angles) is updated in each frame with the sensor estimations made during a person walking 180° around the robot's head. Streams (**a**) and (**b**) are obtained with an speaker, Stream (**c**) with a quiet person and Stream (**d**) with a person talking, but placed under the field of view of the cameras.

Another three tests were conducted with the robot's head moving with the purpose of evaluating the multimodal sensor behavior with dynamically changing conditions. The first test was made twice with two different people walking and talking around the robot during 45 s and in different conditions of luminosity. Figure 9 depicts in streams (a) and (b) the images captured. As can be seen in the two cases, the speaker was the main focus of attention, due to the better performance of the audio localization system tracking people in azimuth degrees close to the central bin. The second test was made with the goal of analyzing whether or not the multimodal sensor may also take advantage of the visual and audio systems benefits, separately. Thus, this test was made with a person walking around the robot without talking (Stream c)) and with a person talking, but not being visible to the robot's head (Stream d)). In both cases, the system was able to follow the person, but the tracked person was not always the center of the image, as in the multimodal sensor streams, which indicates a lower accuracy.

As pointed out by Nakamura *et al.* [6], the multimodal sensor was able to compensate for the limitations of auditory and visual tracking, as well as improving the tracking accuracy. Moreover, the implementation presented here was also able to exploit not only azimuth angle detection, but also the information of the elevation angles extracted from the segmentation algorithm. These results confirm that the bio-inspired multimodal sensor described is able to detect a speaker overcoming possible uncertainty due to changing conditions. Indeed, it is able to combine audio and visual information without loosing the strengths that visual and auditory systems have separately. That is to say, avoiding the typical false positive detection of visual systems once the person is speaking and being able to follow a quiet person.

Ferreira *et al.* [17] presented a similar attempt to evaluate the behavior of a multimodal sensor mounted on a robot's head for real-time conditions and in a two-speaker scenario, via an annotated timeline. The scenario consisted of a lab with two people placed approximately between 1 or 2 m from the robot's head and within azimuth angles of 45° and 135°, therefore not finding difficulties with the face-detector implemented with the OpenCV library. In particular, they also reported what can be considered as proper behavior by fixing the robot's head to both speakers when they were speaking or changing their positions, demonstrating that the fusion of information had more advantages than just a pure “sum of parts”. However, they also highlighted the need to select proper weights for the beta distributions of the active perception hierarchy used to adapt the robot's behavior to different interaction

scenarios. Moreover, they also could test the possibility of tracking one person as long as he/she walked within reasonable velocity limits. Our results also indicate the feasibility of using a multimodal sensor for tracking purposes on a wider range, although for just a single speaker. For distances up to 6 m, but with a more controlled scenario (empty lab and corridors), Nickel and Stiefelhagen [18] also presented the feasibility of using a fast audio-visual multi-person tracking system for a humanoid robot. However, although the tracker showed a solid performance, it also reported track loss due to periods of the person's inactivity at far distances or in a turned-away position. Indeed, it seems that the use of a dense fusion algorithm of too many features (13 visual cues, although the experiments were made with a reduced set, and the use of an array of microphones) limited the experiments to a camera motion controlled by a human operator.

**Figure 10.**
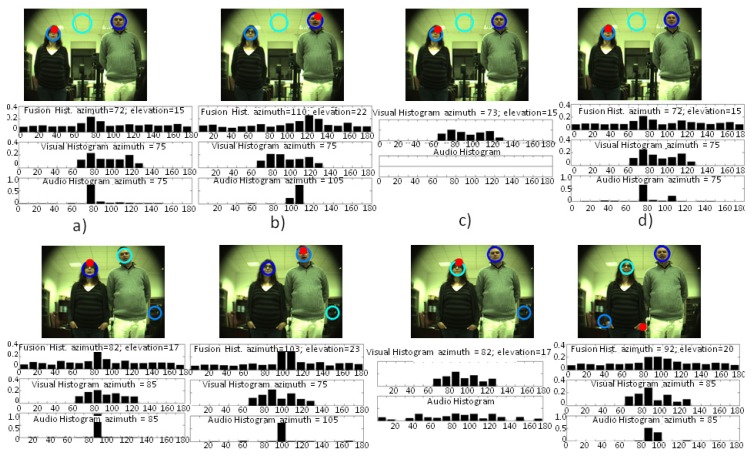
Results obtained with two speakers placed at two different distances between them. (**a**) The person on the left talking. (**b**) The person on the right talking. (**c**) None of the people talking. (**d**) Two people talking simultaneously. The multimodal sensor estimation is marked with a red circle. Blue circumferences show OpenCV face-detector estimations.

In Figure 10, the histograms computed by the three systems (audio localization, visual localization and multimodal sensor) and images with the multimodal sensor estimation (red circle) with two people and two different separations between them can be seen. As can be seen in Figure 10d, the results with the two people speaking simultaneously indicate the feasibility of detecting both by just combining the two peaks found in the histograms computed for visual and audio localization systems. In this case, the multimodal sensor that was trained for just one speaker was detecting in one case the left speaker and in the other case (bottom results) the proto-object with higher saliency (the hand) in an intermediate position between the speakers. As in the work of Asano *et al.* [13], exploiting a Bayesian network to fuse visual and audio cues seems to be a promising tool for the fast tracking of speakers. Indeed, the evidence histogram approach would allow multi-speaker tracking, at least with a reduced number of speakers (with just two microphones), via exploiting the detection of the multiple peaks shown in the histograms of Figure 10d.

The results of this last test also show the feasibility of using the multimodal sensor when two people were present in a scenario where just one speaker was expected. As can be seen in the results depicted in Figure 10a, the speaker on the left was tracked properly by the three systems. The histogram results of Figure 10b show how, for both cases, the visual system marked with higher saliency the person on the right. It can be seen how the audio system was localizing the person within the bins of 105° (upper image) and 95° (bottom image), while the multimodal fusion was able to localize the speaker at 110° and 103°. Thus, it was then able to correct slightly the lack in resolution of the audio localization system. The results of Figure 10c also showed how the multimodal sensor takes advantage of the visual estimation when the audio information is not relevant.

## 6. Conclusions and Future Work

This research study presents a method for detecting and tracking a speaker based on a multimodal sensor. In particular, the approach followed to implement this sensor consists of fusing visual and audio evidence about the presence of a speaker via using a Bayes network. The bio-inspired approach is exploited in two ways. First, by modeling the human learning process with a training process employed to build up a maximum *a posteriori* matrix, by which states and observations are associated, for the particular case of locating a speaker placed in the surroundings. Second, the multimodal sensor is built by combining the information obtained from just two video cameras and two microphones, as humans do, instead of using more complex and computationally demanding approaches (with a higher number of microphones or other types of sensors, such as depth cameras, thermal cameras, *etc*.)

The experiments performed to evaluate this multimodal sensor have probed its reliability in overcoming the typical limitations of tracking systems while working in interactive time and in real conditions, such as dealing with cluttering spaces, luminosity changes, environmental noise, *etc*. Indeed, the integration via a simple Bayes network has allowed for exploiting the benefits that visual and audio localization systems present separately and to contribute to arising performance. Thus, this sensor is able to focus attention on a speaker placed outside the camera's field of view and to increase the possible lack of resolution in identifying a speaker just in the horizontal plane and within ranges of 10°.

The developed system may enable robots to track a person in daily-life environments by following a bio-inspired approach featured by its low demand of resources. Moreover, the system is ready to incorporate mechanisms that would allow it to track multi-speakers by exploiting the characterization made of the evidence probability through the histograms. Indeed, as future research, we are already working on incorporating algorithms to detect multiple speakers without the need of knowing *a priori* the number of active sources.

As described, Nerve is the middleware used to communicate the different modules that implement the multimodal sensor. Indeed, the multimodal sensor implemented is being used also as a testbed to improve the dynamic capabilities of Nerve, by allowing it to auto-reconfigure itself in order to reach the best performance. The idea is to deal effectively with the more than twenty quality of services parameters that DDS has. Thus, in a typical situation for a robot that interacts with people in which the presence of a person is detected, the system could prioritize the multimodal sensor qualities of service (decision times, audio and visual histograms resolutions, noise detection, *etc.*), while keeping the other parameters at the optimal level to achieve the highest possible performance (memory consume, data transfer, *etc.*).

More exhaustive tests must be also done before this multimodal sensor is incorporated on a attentional mechanism with general purposes, that is to say, able to dynamically adapt its behavior depending on the task being performed.
